# Sonodynamic Therapy With Anticancer Micelles and High-Intensity Focused Ultrasound in Treatment of Canine Cancer

**DOI:** 10.3389/fphar.2019.00545

**Published:** 2019-05-21

**Authors:** Yuki Horise, Masanori Maeda, Yoshiyuki Konishi, Jun Okamoto, Soko Ikuta, Yoshiharu Okamoto, Hiroshi Ishii, Shin Yoshizawa, Shinichiro Umemura, Tsuyoshi Ueyama, Satoshi Tamano, Atsushi Sofuni, Kazuhisa Takemae, Ken Masamune, Hiroshi Iseki, Nobuhiro Nishiyama, Kazunori Kataoka, Yoshihiro Muragaki

**Affiliations:** ^1^Institute of Advanced Biomedical Engineering and Science, Tokyo Women's Medical University, Tokyo, Japan; ^2^MakeWay Merger Company, Saitama, Japan; ^3^Faculty of Agriculture, Tottori University, Tottori, Japan; ^4^Tokyo Animal Medical Center, Tokyo, Japan; ^5^Department of Communications Engineering, Tohoku University, Sendai, Japan; ^6^Department of Biomedical Engineering, Tohoku University, Sendai, Japan; ^7^Medical Business Department, DENSO Corporation, Nisshin, Japan; ^8^Healthcare Business Unit, Hitachi, Ltd, Tokyo, Japan; ^9^Department of Gastroenterology and Hepatology, Tokyo Medical University Hospital, Tokyo, Japan; ^10^Pharmaceutical Division, Kowa Company, Ltd, Tokyo, Japan; ^11^Polymer Chemistry Division, Tokyo Institute of Technology, Meguro, Japan; ^12^Department of Materials Engineering, The University of Tokyo, Tokyo, Japan

**Keywords:** sonodynamic therapy, minimally invasive, drug delivery system, high-intensity focused ultrasound, anticancer micelles

## Abstract

Sonodynamic therapy (SDT) is a minimally invasive anticancer therapy involving a chemical sonosensitizer and high-intensity focused ultrasound (HIFU). SDT enables the reduction of drug dose and HIFU irradiation power compared to those of conventional monotherapies. In our previous study, mouse models of colon and pancreatic cancer were used to confirm the effectiveness of SDT vs. drug-only or HIFU-only therapy. To validate its usefulness, we performed a clinical trial of SDT using an anticancer micelle (NC-6300) and our HIFU system in four pet dogs with spontaneous tumors, including chondrosarcoma, osteosarcoma, hepatocellular cancer, and prostate cancer. The fact that no adverse events were observed, suggests the usefulness of SDT.

## Introduction

Sonodynamic therapy (SDT) is an anticancer therapeutic approach that has the potential to non-invasively eradicate solid tumors in a site-directed manner via cytotoxicity induced by the combination of a chemical sonosensitizer and high-intensity focused ultrasound (HIFU) (McHale et al., 2016). It is thought that interactions between the sonosensitizer and ultrasound generate reactive oxygen species (ROS), including singlet oxygen and hydroxyl radicals, which kill targeted cancer cells via apoptosis and necrosis (Dellinger, 1996; Oleinick et al., 2002), and studies on ROS generation have been carried out (Yumita et al., 2014; Giuntini et al., 2018). Similarly, photodynamic therapy (PDT), in which a cytotoxic effect is induced via the interaction between a photosensitizer and light of a specific wavelength, has a similar mechanism of action to SDT and has been used clinically in the treatment of lung cancer and brain tumors (Kato, 1998; Dolmans et al., 2003; Moghissi et al., 2007). However, while PDT is only useful for superficial tumors due to low penetrance of light into deeper tissues, SDT based on HIFU can be used for non-dermatological cancers that lie within ultrasound range (Ji et al., 2009). Several SDT studies using various sonosensitizer drugs have recently been reported. In general, the combination of doxorubicin and HIFU has been frequently reported as a popular SDT (Umemura et al., 1997). Suehiro et al. proposed an SDT method involving the use of 5-aminolevulinic acid (5-ALA) for malignant gliomas, and cytotoxicity toward brain tumors of mice was confirmed in glioma cells *in vitro* (Suehiro et al., 2018). In another study using 5-ALA, the anticancer effect of SDT with shock wave was investigated in rat breast cancer model (Foglietta et al., 2015). An SDT-based treatment with Rose Bengal was implemented in a pancreatic cancer model (BxPc-3) *in vitro* and *in vivo* (McEwan et al., 2015), and the apoptosis-promoting effects and mechanism of SDT using hematoporphyrin monomethyl ether (HMME) were examined for the treatment of endometrial cancer *in vitro* (Sun et al., 2015).

The testing of newly developed cancer drugs in canines is common. While drug testing in rodent models presents several limitations in terms of sample collection, surgical interventions, and imaging (Mack, 2005), spontaneous cancers in dogs exhibit strong similarities with human cancers, such as patterns of response or resistance to conventional therapies (LeBlanc et al., 2016). Furthermore, it has been histologically confirmed that various cancers, including osteosarcoma and bladder cancer, are functionally identical in canines and humans (Paoloni et al., 2009; Schiffman and Breen, 2014; Decker et al., 2015), and most human genes have canine orthologs (O'Brien and Murphy, 2003; Lindblad-Toh et al., 2005; Liu et al., 2015; Rogers, 2015). Therefore, human cancer drugs are often tested in pet dogs, albeit at a lower dose according to their size (Ledford, 2016), allowing these dogs to also receive high-quality therapy.

Our proposed SDT adopted an anticancer micelle (NC-6300) as a sonosensitizer. NC-6300 is an antitumor micelle with a diameter of 60–70 nm that incorporates the drug epirubicin (EPI) using micellar nanoparticle technology (Harada et al., 2011). Due to the enhanced permeability and retention (EPR) effect, NC-6300 preferentially accumulates in tumor cells eliciting efficient ROS generation and further increasing the effectiveness of the SDT treatment. This drug has been tested in human clinical trials (Phase I) with advanced or recurrent solid tumors (Mukai et al., 2017). In a previous study involving mouse models of colon cancer (Colon-26) and pancreatic cancer (MIA PaCa-2), we confirmed the improved efficacy of an SDT approach involving the combination of a low dose of NC-6300 and low-energy HIFU relative to the efficacy of NC-6300-only or HIFU-only treatment (Maeda et al., 2017). In order to further the application of our proposed SDT in humans, we conducted a clinical trial of SDT on spontaneous tumors in pet dogs and evaluated the usefulness of our method in an environment similar to a clinical setting.

## Materials and Methods

### Animals

All therapies were performed under the guidelines of the Animal Use Committee of Tottori University and Tokyo Women's Medical University. The protocol was approved by the Committee on the Ethics of Animal Experiments of Tottori University (Approval Number: H27-001) and Tokyo Women's Medical University (Approval Number: AE16-143). Before treatment, informed consent was obtained from the guardians of the animals.

Subject recruitment at Tottori University and Tokyo Women's Medical University, were undertaken by staff veterinarians, who proposed SDT to animal guardians at outpatient clinics. Subjects were recruited to the study if the standard treatment (surgical resection, radiation therapy, or chemotherapy) was impossible or undesirable to the animal guardians. Accordingly, four dogs were enrolled in the study and five SDT sessions were implemented, two sessions to subject A, and one to each of subjects B, C, and D. The weight, gender, and age of each subject at recruitment are indicated in Table 1A. All subjects were admitted to the animal hospital prior to SDT and kept under observation for 2 days. Once the dogs' performance was recovered, as measured by gait analysis and appetite, they were discharged from the hospital.

**Table 1:** Information on study subjects and SDT procedures used for treatment.

**(A)**						
**ID**	**Breed**	**Age**	**Gender**	**Weight (kg)**	**Tumor type**	**Tumor location**
A	Miniature Schnauzer	12	Male	7.5	Chondrosarcoma	Pars pelvica
B	Golden Retriever	8	Male	32.0	Osteosarcoma	Distal right thigh
C	Yorkshire Terrier	13	Male	3.5	Hepatocellular cancer	Liver
D	French Bulldog	11	Male	13.5	Prostate cancer	Prostate gland
**(B)**						
**Session**	**ID**	**Tumor type**	**NC-6300 dosage (mg/m**^**2**^**)**	**Number of HIFU sequences**	**Survival period after SDT (months)**	
1	A	Chondrosarcoma	7.5	9	25	
2	A	Chondrosarcoma	15.0	17	23	
3	B	Osteosarcoma	15.0	22	5	
4	C	Hepatocellular cancer	30.0	20	10	
5	D	Prostatic cancer	30.0	30	4	

### Sonosensitizer (NC-6300)

**Figure 1:**
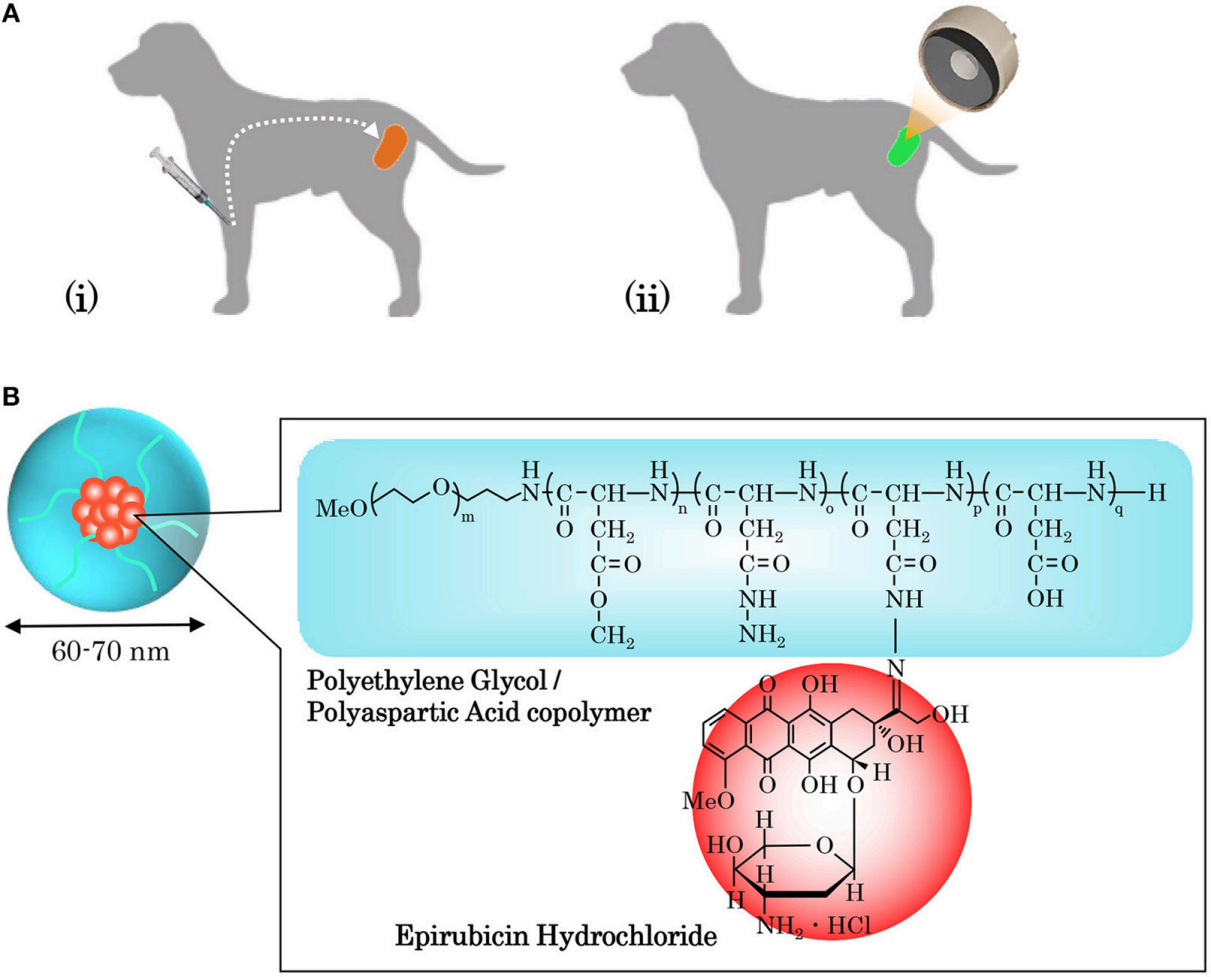
Operating process of sonodynamic therapy (SDT) and combined application of a sonosensitizer drug (NC-6300). **(A)** SDT combines the use of high-intensity focused ultrasound (HIFU) and a chemical sonosensitizer, and their interaction generates reactive oxygen species (ROS), which kill targeted cancer cells via apoptosis and necrosis. To implement SDT, (i) the sonosensitizer is administered intravenously to a patient, and (ii) 24 h later, HIFU irradiation of the targeted tumor is conducted. **(B)** Our proposed SDT adopted NC-6300 as a sonosensitizer. NC-6300 is an anticancer agent comprised of epirubicin (EPI), that is covalently bound to polyethylene glycol polyaspartate block copolymers through an acid-labile hydrazone bond, in a nano-micellar structure with a diameter of 60–70 mm. NC-6300 preferentially accumulates in tumor cells due to the enhanced permeability and retention (EPR) effect, and thus the targeted tumor can be intensively treated.

As shown in Figure 1, NC-6300 was diluted to an arbitrary concentration with phosphate-buffered saline (PBS) and was injected intravenously into patient dogs 24 h before HIFU. It was reported by Bae et al. that the highest accumulation of the micelle occurred 24 h after administration (Bae et al., 2005). Therefore, the therapeutic modality adopted in our study was in line with our previous experiments with mouse models (Maeda et al., 2017), in which the drug was administered 24 h prior to HIFU irradiation. The drug dose was based on the body surface area (BSA), which was calculated from body weight (BW) using a dose conversion table for dogs (Saganuwan, 2017).

For conventional EPI treatment, Nagata et al. previously reported a toxicity test for beagles that indicated a 50% lethal dose (LD50) in male and female beagles of 2.25 (2.07–2.49) mg/kg and 2.59 (2.42–2.91) mg/kg, respectively (Nagata et al., 1986). In another study by Marrington et al., the toxicity associated with EPI treatment was investigated in 139 dogs of various breeds, and most of these dogs received a dose of 30 mg/m^2^ (Marrington et al., 2012). Further, a study conducted by Kowa Company administered NC-6300 at 30 or 45 mg/m^2^ to beagles, while the control group received conventional EPI at 30 mg/m^2^ (unpublished data). Accordingly, the results indicated that NC-6300 at 30 mg/m^2^ was safer than conventional EPI at 30 mg/m^2^. Using 45 mg/m^2^ NC-6300, there were no serious adverse events, although recovery of cells, such as leukocytes, from hematotoxicity tended to be delayed relative to that in cases treated with the lower dose. In all cases, transitory changes in systemic flushing and edema were observed immediately after drug administration and disappeared within 1 h. Based on the results of this toxicity test in beagles, the dose per unit area of the drug and concentration of the infusion fluid were assumed as safe for use in this study. The NC-6300 dose in session 1 (7.5 mg/m^2^) was calculated from the dose (2.5 mg/kg) used in previous experiments with mouse models (Mukai et al., 2017) that was based on an FDA guidance (FDA, 2005). We gradually increased the dose of NC-6300 while examining its safety at each session.

### HIFU System

**Figure 2:**
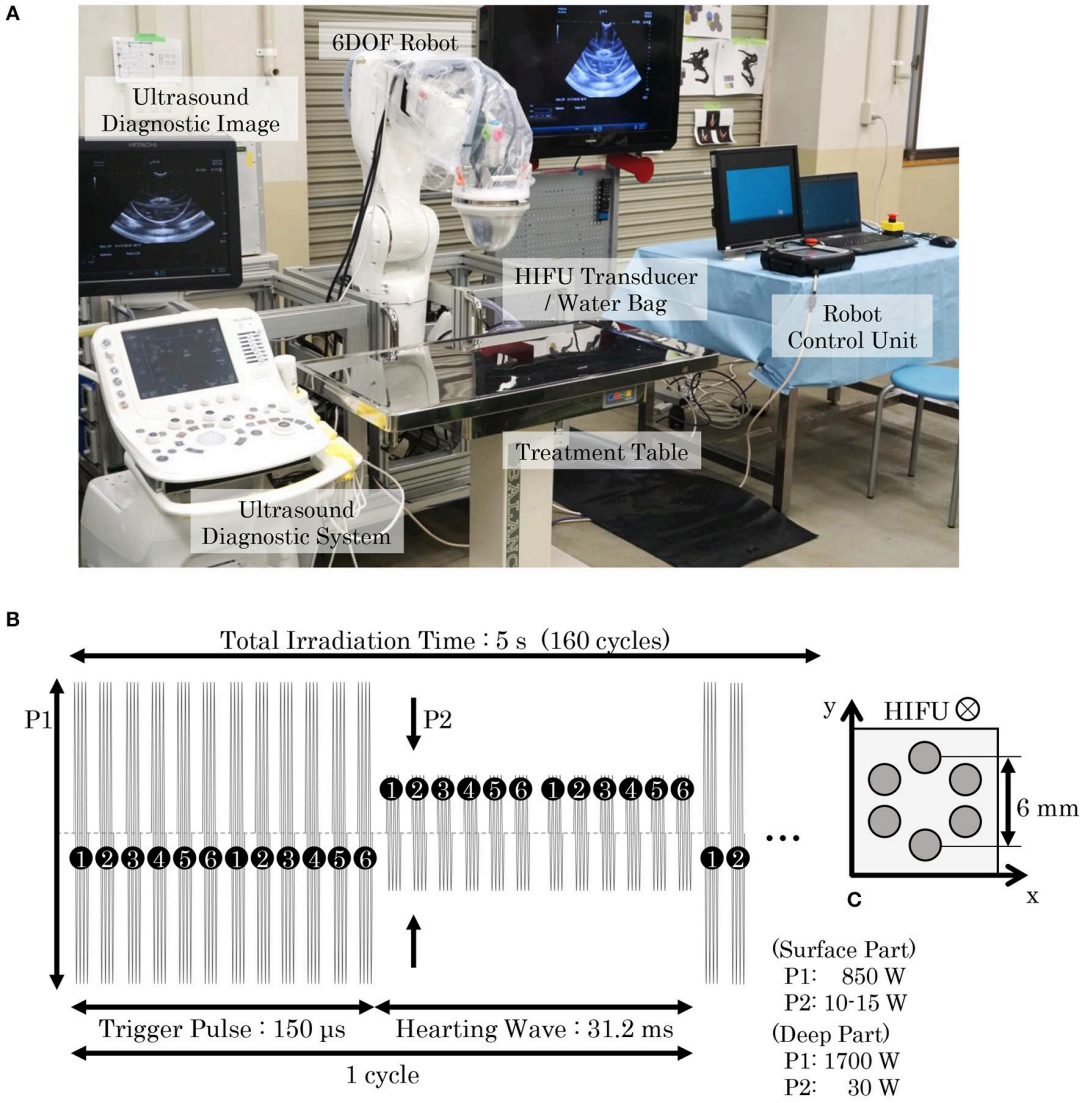
HIFU system and HIFU irradiation sequence. **(A)** Our HIFU system consisted of a HIFU-irradiation system, a diagnostic imaging system, and a robot control system. A HIFU transducer was attached to the distal part of a 6-DOF robot and covered with a water bag filled with degassed water to avoid ultrasound attenuation. An ultrasound probe was set in the central axis of the HIFU transducer, allowing the ultrasonic image to be observed with the ultrasound diagnostic system. The placement of the HIFU transducer can be robotically arranged using a dedicated robot control unit. When conducting SDT for pet dogs, they were anesthetized and laid on a treatment table. An operator moved the HIFU transducer and set the target position while observing the ultrasound image, and then initiated the HIFU irradiation. **(B)** In our proposed SDT, two types of irradiation patterns for trigger and heating were combined, with the irradiation cycle repeating 160 times in 5 s. **(C)** In a single irradiation sequence, six points arranged in a 6-mm circle were irradiated in sequential order. The trigger pulse generated cavitation bubbles, and the heating wave maintained the bubbles to enhance the cell-killing effect. The output powers of the trigger pulse and the heating wave were determined by the depth of the irradiation target.

The HIFU system was composed of a HIFU transducer (IMASONIC SAS, Voray-sur-l'Ognon, France), a water bag, a 6-DOF robot (VS087, DENSO WAVE Inc., Aichi, Japan), a dedicated robot control unit, and an ultrasound diagnostic system (ARIETTA 60, HITACHI Ltd., Tokyo, Japan), as shown in Figure 2A. The HIFU transducer, which has a spherical surface and coaxial cylinder, was attached to the distal part of the robot and covered with a water bag containing degassed water. A diagnostic probe (electric convex probe C22K; HITACHI Ltd., Tokyo, Japan) was inserted into the center of the HIFU transducer in order to visualize the inside of the body using the ultrasound diagnostic system. The probe can be translated coaxially up to a maximum distance of 120 mm from the irradiation surface of the transducer. The irradiation targeting point can be easily set using the 6-DOF robot controlled by the robot control unit while observing the ultrasound diagnostic image in real time.

The HIFU transducer was an array-type piezo-composite transducer (resonant frequency: 1 MHz, focal length: 120 mm, outer diameter: 120 mm, inner diameter: 40 mm) energized by a radiofrequency amplifier (Class D amplifiers, MICROSONIC Co., Tokyo, Japan). A function generator (WF1974; NF Co., Kanagawa, Japan) generated the trigger signal to the amplifier and was controlled by software we developed. As illustrated in Figure 2B, the electric sequence of the HIFU irradiation consisted of two waveforms: (1) a trigger pulse for generating cavitation bubbles, and (2) a heating wave for bubble-enhanced heating. One HIFU irradiation sequence took 5 s (160 cycles), and six targeting points were heated in order, as shown in Figure 2C. The output power of the trigger pulse and heating wave (P1 and P2) were selected according to the depth of the targeting tumor: at a depth ≥2 cm, the point was regarded as being “deep,” and P1 and P2 were thus set to 1,700 and 30 W respectively; otherwise at depths < 2 cm, P1 and P2 were set at 850 W and 10–15 W, respectively. The HIFU intensity was adjusted accordingly to previous experiments with mouse models (Maeda et al., 2017) and the effect of attenuation on HIFU-treated tissues was considered. The size of one focal spot of the HIFU was approximately 3 mm in diameter and 8 mm in length.

In preparation for HIFU, the hairs around the irradiated site were shaved to prevent bubble generation, which could cause burn injury. After identifying the exact location of the tumor using a conventional diagnostic probe, the HIFU transducer covered with the water bag was placed in the appropriate position, and ultrasound gel was applied between the body surface and the water bag. The number of HIFU irradiation sequences was dependent on the size of the targeting tumor and the passing rate of the ultrasound. To confirm the safety of SDT, the irradiated region was limited to low-risk irradiation, instead of whole tumor irradiation. In particular, areas with potential appearance of serious adverse events such as large vessels or the area around those and the bones where unexpected ultrasound reflections may occur, were avoided.

### Outcomes

For hematological examination 10 parameters were assessed, red blood cell count, white blood cell count, hemoglobin, hematocrit, mean cell volume, mean cell hemoglobin, mean cell hemoglobin concentration, platelet count, platelet distribution width, and mean platelet volume. Biochemical parameters included albumin, alanine transaminase, aspartate transaminase, alkaline phosphatase, gamma glutamyl transferase, total bilirubin, total cholesterol, glucose level, blood urea nitrogen, creatinine, Ca, Na, K, and Cl. Blood was collected before NC-6300 administration and on the day of HIFU irradiation. Additional hematological and biochemical examinations were implemented every few days after the therapy.

Tumor size was measured using 2D images that were acquired using CT, MRI, US, and X-ray. A veterinarian visually conducted the gait evaluation; common performance evaluation methods had not yet been established, thus we assessed the status of subjects with regards to gait, appetite, excretory function, and blood parameters.

## Results

SDT was conducted on four pet dogs (subjects A, B, C, and D) with spontaneous tumors (chondrosarcoma, osteosarcoma, hepatocellular cancer, and prostate cancer); the age and weight of subjects when SDT was performed are indicated in Table 1A. HIFU irradiation was conducted 24 h after intravenous administration of an anticancer micelle (NC-6300). These therapies were administered at Tottori University and Tokyo Women's Medical University in accordance with the circumstances of the animal guardians and attending veterinarians. The method of anesthesia during therapy was dependent on each facility's anesthesiologist. For subjects A and B treated at Tottori University, butorphanol and midazolam were administered as pre-anesthetic agents; general anesthesia was induced with intravenous propofol and maintained with isoflurane and oxygen after tracheal intubation. For subjects C and D, treated at Tokyo Women's Medical University, atropine and buprenorphine were administered as pre-anesthetic agents; general anesthesia was induced with intravenous propofol and maintained with isoflurane.

Five SDT sessions were performed in the four dogs under the treatment conditions indicated in Table 1B. For subject A, a second SDT session was initiated 2 months after the first session. For all SDT sessions, no adverse events occurred during drug administration or HIFU irradiation, and no abnormalities were observed based on hematological and biochemical tests although temporary changes in some parameters such as WBC and GLU were noticed, as indicated in Tables 2–6. In addition, there were no major changes in terms of body weight or food consumption after therapy relative to the baseline.

**Table 2:** Results of the hematological and biochemical test in session 1.

**Examination item**		**Day −1**	**Day 1**	**Day 7**
RBC	[10^4^/μl]	616	532	543
WBC	[10^2^/μl]	69	76	178
HGB	[g/dl]	14.3	12.2	12.4
HCT	[%]	38.2	32.8	33.3
MCV	[fL]	62	61.7	61.3
MCH	[pg]	23.2	22.9	22.8
MCHC	[g/dL]	37.4	37.2	37.2
PLT	[10^4^/μl]	62	57	58
PDW		10.7	9.6	11
MPV	[fl]	9.2	8.9	10.2
ALB	[g/dl]	NA	3.3	3.2
ALT	[U/l]	60	56	43
AST	[U/l]	36	46	40
ALP	[U/l]	338	313	287
GGT	[U/l]	10	9	7
TBil	[mg/dl]	0.1	0.2	0.3
TCho	[mg/dl]	419	328	348
GLU	[mg/dl]	116	119	118
BUN	[mg/dl]	15.6	18.4	14.9
CRE	[mg/dl]	0.6	1.8	0.5
Na	[mmol/l]	147	150	144
K	[mmol/l]	4.3	4.2	4.4
Cl	[mmol/l]	109	114	104

**Table 3:** Results of the hematological and biochemical test in session 2.

**Examination item**		**Day −1**	**Day 1**	**Day 3**
RBC	[10^4^/μl]	626	609	622
WBC	[10^2^/μl]	50	71	59
HGB	[g/dl]	14.4	13.9	14
HCT	[%]	38.3	37.4	38
MCV	[fL]	61.2	61.4	61.1
MCH	[pg]	23	22.8	22.5
MCHC	[g/dL]	37.6	37.2	36.8
PLT	[10^4^/μl]	58	54	61
PDW		10.1	9.8	10.1
MPV	[fl]	9.2	9	9.4
ALB	[g/dl]	3.6	3.4	3.6
ALT	[U/l]	55	52	55
AST	[U/l]	33	53	47
ALP	[U/l]	447	443	415
GGT	[U/l]	6	8	6
TBil	[mg/dl]	0.3	0.2	0.3
TCho	[mg/dl]	>450.0	423	>450.0
GLU	[mg/dl]	130	122	132
BUN	[mg/dl]	16.7	20.5	20.4
CRE	[mg/dl]	1.4	0.8	1.6
Na	[mmol/l]	147	NA	150
K	[mmol/l]	4.9	NA	5.2
Cl	[mmol/l]	109	NA	110

**Table 4:** Results of the hematological and biochemical test in session 3.

**Examination item**		**Day −1**	**Day 1**	**Day 6**
RBC	[10^4^/μl]	756	630	660
WBC	[10^2^/μl]	82	66	92
HGB	[g/dl]	16.8	13.9	13.6
HCT	[%]	47	38.6	43.5
MCV	[fL]	62.2	61.3	65.9
MCH	[pg]	22.2	22.1	20.6
MCHC	[g/dL]	35.7	36	31.2
PLT	[10^4^/μl]	49	38	30
PDW		9.5	9.4	15.2
MPV	[fl]	8.3	8.8	8.8
ALB	[g/dl]	2.9	2.4	2.4
ALT	[U/l]	36	33	80
AST	[U/l]	46	28	32
ALP	[U/l]	355	456	793
GGT	[U/l]	7	5	11
TBil	[mg/dl]	0.5	0.4	0.3
TCho	[mg/dl]	441	417	426
GLU	[mg/dl]	106	117	74
BUN	[mg/dl]	17.7	15.1	24
CRE	[mg/dl]	1.2	0.7	0.7
Na	[mmol/l]	148	NA	150
K	[mmol/l]	4.4	NA	5
Cl	[mmol/l]	118	NA	112

**Table 5:** Results of the hematological and biochemical test in session 4.

**Examination item**		**Day −6**	**Day 1**	**Day 6**
RBC	[10^4^/μl]	647	706	654
WBC	[10^2^/μl]	87.4	94.5	85.8
HGB	[g/dl]	14.4	13.9	14.4
HCT	[%]	41.7	45.8	42.1
MCV	[fL]	64.5	64.9	64.4
MCH	[pg]	22.3	22.1	22
MCHC	[g/dL]	34.5	34.1	34.2
PLT	[10^4^/μl]	49.6	60.6	56.2
ALB	[g/dl]	3.6	3.85	3.38
ALT	[U/l]	365	331	282
ALP	[U/l]	226	266	231
GLU	[mg/dl]	86	94	92
BUN	[mg/dl]	37	44	22
CRE	[mg/dl]	0.8	1	0.7
Na	[mmol/l]	155.6	152.3	NA
K	[mmol/l]	4.74	4.16	NA

**Table 6:** Results of the hematological and biochemical test in session 5.

**Examination item**		**Day −1**	**Day 1**	**Day 7**
RBC	[10^4^/μl]	650	527	548
WBC	[10^2^/μl]	13.9	17.6	11.7
HGB	[g/dl]	15.8	12.6	14.1
HCT	[%]	46.4	NA	NA
MCV	[fL]	71.4	69.8	70.6
MCH	[pg]	24.3	23.9	25.7
MCHC	[g/dL]	34.1	34.2	36.4
PLT	[10^4^/μl]	67.8	67.3	49.4
ALB	[g/dl]	2.9	NA	NA
ALT	[U/l]	44	55	76
AST	[U/l]	35	120	29
ALP	[U/l]	276	NA	NA
GLU	[mg/dl]	106	116	NA
BUN	[mg/dl]	10.1	11.1	14.4
CRE	[mg/dl]	1	0.7	0.7

**Figure 3:**
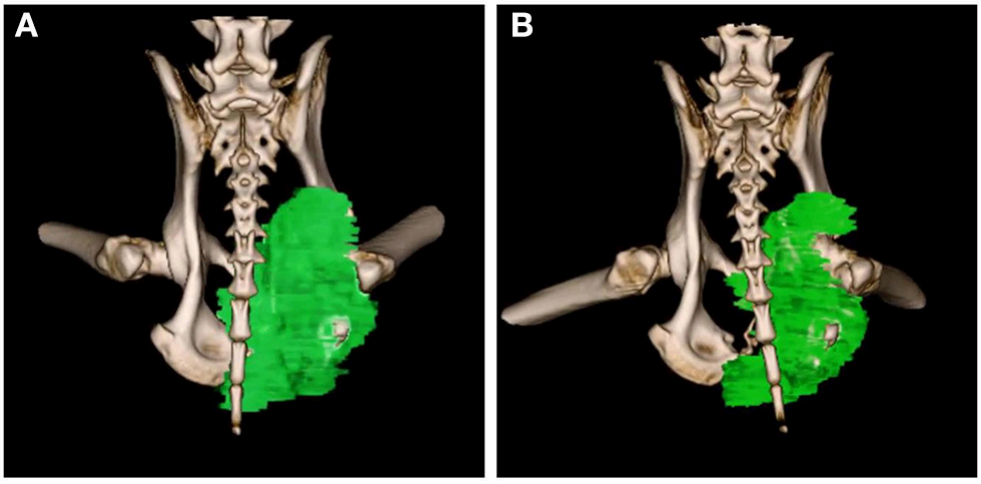
3D-CT images before and after SDT (session 1). In session 1 for treatment of subject A with a chondrosarcoma, 3D-CT images were taken before and after SDT. The tumor size was measured before SDT **(A)**, and the volume was reduced by about 15% compared to that in the CT image taken 2 weeks after SDT **(B)**.

Upon subject A's first visit at 10 years old, an enlargement in the right hip was diagnosed as chondrosarcoma derived from the pelvis. Although therapies such as ablation and cryotherapy were performed previously, SDT was selected as a new approach due to the cancer's recurrence. Following SDT session 1, the tumor size was 48.8 × 32.2 mm 2 weeks before SDT and 47.7 × 29.2 mm 1 week post-SDT. The tumor size was reduced to 85% according to a CT image taken 2 weeks after SDT (Figure 3). In addition, walking was markedly improved (Video S1). Before SDT, subject A's right posterior leg shook, and standing on all four legs was difficult due to pain. By contrast, after SDT, subject A was able to walk, and even run, steadily using all four legs. As the subject's physical condition was stable after the first SDT session, a second round of SDT (session 2) was performed 2 months later. As unexpected clinical effects, such as improvement in walking, were observed with no adverse events after SDT (session 1), the number of irradiations and the drug dose were increased in session 2 accordingly. Following session 2, the tumor size was 54.9 × 31.71 mm before SDT and 52.4 × 30.81 mm 1 week post-SDT, and walking was the same as that after session 1. However, subject A's excretory function returned to normal, and there was only a minimal increase in tumor volume at follow-up 4 months later.

Subject B exhibited claudication of the left posterior leg and was diagnosed with osteosarcoma. Following a single SDT session (session 3), the tumor size was 1.46 × 1.42 mm before SDT and 1.29 × 1.36 mm on the 11th day post-SDT. The subject's walking gait was improved 10 days post-SDT (Video S2), and the strength of analgesic was reduced compared with that administered before treatment. At the 3-month follow-up, the severity of pain had not worsened compared to that before treatment.

**Figure 4:**
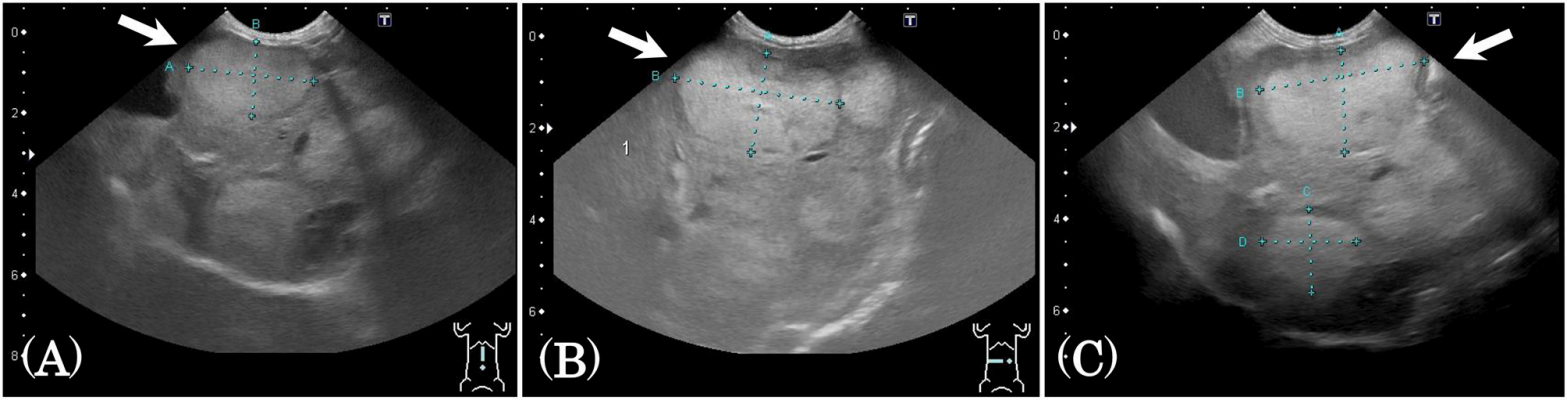
Ultrasound images before and after SDT (session 4). In session 4, treatment of subject C with hepatocellular cancer, ultrasound images were observed and compared before and after treatment. **(A)** The tumor size was 18.3 × 31.2 mm 9 days before SDT, **(B)** 21.8 × 36.4 mm 1 day before SDT, and **(C)** 22.2 × 36.5 mm 6 days after SDT. The growth rate of the tumor was reduced, as demonstrated by the fact that the tumor size increased 1.39-fold over 8 days before SDT and only 1.02-fold over 7 days after treatment.

Subject C was diagnosed with hepatocellular cancer, and surgery was performed 10 months before SDT. However, recurrence was observed, with tumor growth as shown in Figure 4; tumor size was 18.3 × 31.2 mm 9 days before SDT, and 21.8 × 36.4 mm 1 day before SDT, representing a 1.39-fold increase. Following SDT (session 4), tumor size was 22.2 × 36.5 mm at 6 days post-SDT, representing a 1.02-fold increase over the tumor size 1 day before SDT (Figure 4). From this result, the growth rate of the tumor in the HIFU-irradiated area was found to have retarded compared to the growth rate before treatment.

**Figure 5:**
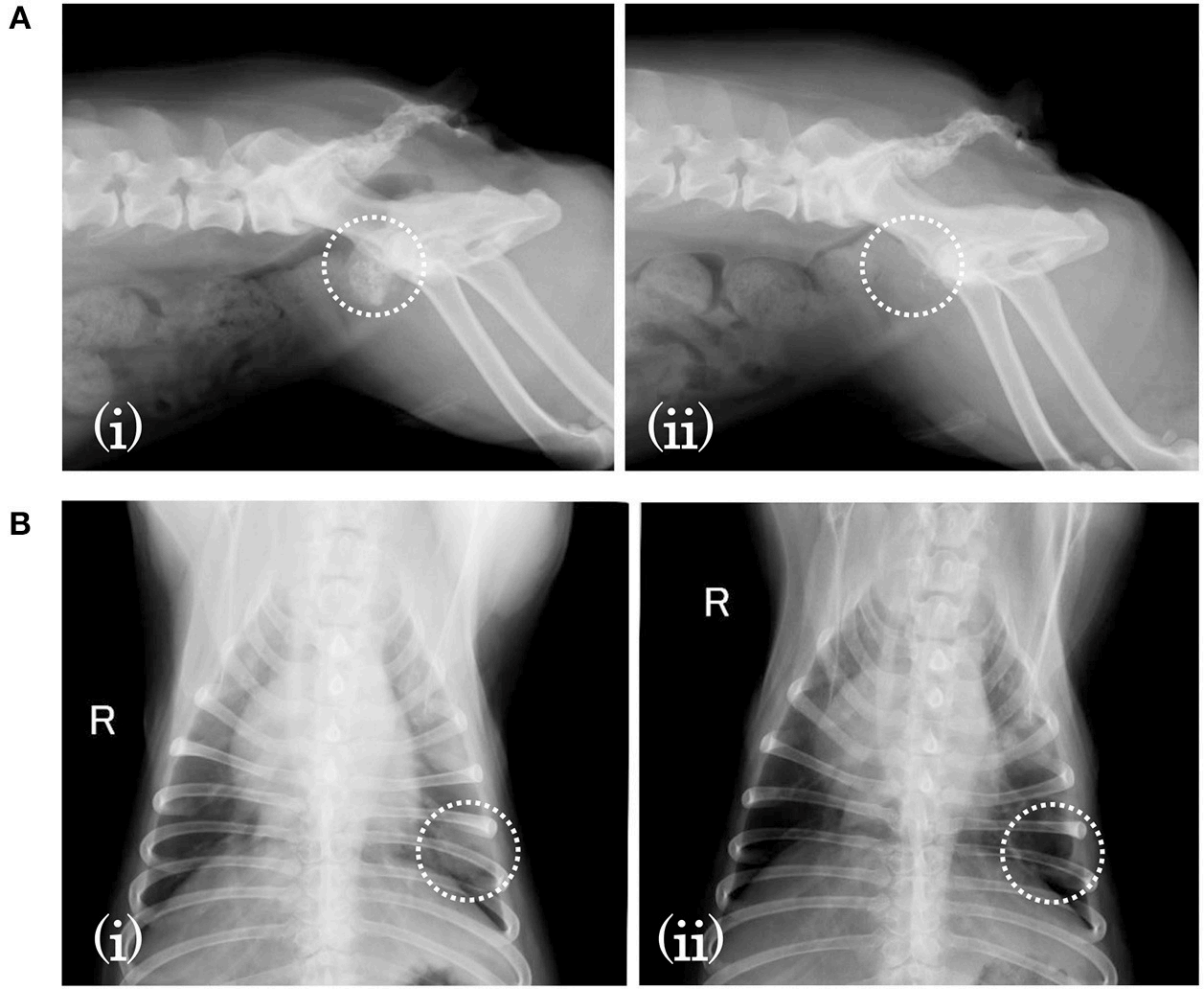
X-ray images before and after SDT (session 5). In session 5, treatment of subject D with prostate cancer and lung involvement, **(A)** a calcified mass was observed inside the tumor 18 days before SDT (i) but had disappeared by 49 days after treatment (ii). **(B)** As for the involved lung cancer, it was observed 18 days before SDT (i) but had vanished by 49 days after SDT (ii).

Subject D was diagnosed with prostate cancer, and a calcified mass was observed inside the tumor in an X-ray taken 18 days before SDT. The subject also developed metastatic lung cancer. Following SDT (session 5), the tumor size was 26.0 × 21.1 mm 1 day before SDT and 35.1 × 23.3 mm on 3 months post-SDT. The urinary frequency of the subject was improved, and the calcified mass inside the tumor disappeared by 49 days post-SDT, as indicated in Figure 5A. Moreover, a metastatic lung tumor also disappeared based on radiographic imaging, as shown in Figure 5B.

The information pertaining to tumor size before and after SDT in all the five sessions is arranged in Table 7.

**Table 7:** Tumor size before and after SDT in all the five sessions.

**Session**	**Tumor size before SDT (mm^**2**^)**	**Tumor size after SDT (mm^**2**^)**
1	48.8 × 32.2	47.7 × 29.2
2	54.9 × 31.71	52.4 × 30.81
3	1.46 × 1.42	1.29 × 1.36
4	21.8 × 36.4	22.2 × 36.5
5	26.0 × 21.1	35.1 × 23.3

## Discussion

Our SDT method combines an anticancer micelle (NC-6300) and a novel HIFU system. This treatment is assumed to be both effective and safe, enabling reductions in drug dose and HIFU irradiation power compared with those of conventional single therapies. The effectiveness of the proposed SDT was demonstrated previously in mouse models of colon and pancreatic cancer. In the current study, we evaluated the usefulness of our SDT approach in pet dogs to enable future clinical application. Although HIFU is considered to cause adverse events, such as burn injury, vascular obstruction, hematoma, and aching pain (Illing et al., 2005; Furusawa et al., 2007; Sofuni et al., 2014), no adverse events following HIFU irradiation were observed in any of our canine SDT subjects. Furthermore, the drug NC-6300 is thought to potentially cause retinal hemorrhages (Mukai et al., 2017), and EPI hydrochloride, the active substance of NC-6300, is considered to cause serious adverse events such as myocardial disorder, myelosuppression, and anaphylaxis, as well as other adverse events such as cardiac dysrhythmia, hives, abnormal hepatic function (increase in aspartate transaminase [glutamic oxaloacetic transaminase], alanine transaminase [glutamic pyruvic transaminase], etc.), abnormal kidney function (increase in blood urea nitrogen, etc.), and asitia (FDA, 2018). Based on our results from five SDT sessions, there were no adverse events after administration of NC-6300, and no obvious abnormalities were observed following the hematological and biochemical assessment of all subjects; these were considered to be due to the combination of the low dose of NC-6300 and low-energy HIFU. These results indicate that all SDT sessions were conducted safely and that the concentration of the drug and the output power of HIFU were acceptable.

In addition to confirming the safety of our method, the results of SDT further confirmed the anticancer efficacy of this method. For example, following SDT session 1, the ability of subject A to walk was substantially improved. This was presumed to be due to a decrease in pain in the right posterior leg as a result of HIFU, despite the fact that the output power was one order of magnitude lower than that of conventional solo HIFU therapy (Wang et al., 2011; Huisman et al., 2014; Zhang et al., 2016). Moreover, tumor size was reduced by 15%; this was likely due to the multiple effects of SDT, consisting of mechanical destruction by ultrasound, the antitumor activity of NC-6300, and synergistic effects of the dual treatment modality, such as the generation of ROS [hydroxyl and superoxide radicals (data not shown)]. Following SDT session 2, the subject's excretory function improved, again likely as a result of the pain-relieving effects of HIFU. In addition, the size of the tumor increased only slightly over the next 4 months; we speculate that this antitumor efficacy was achieved through the combination of the anticancer NC-6300 micelle and HIFU. Subject A survived for 25 months after SDT session 1 despite severe disease, indicating that subject A survived for approximately 10 “human” years since the age conversion between dogs to humans is considered to be about five times. Thus, this SDT approach is likely to improve prognosis.

In subject B, walking was visibly improved after SDT. As in subject A, we speculate that this was largely due to pain relief in the affected area as a result of HIFU irradiation. In subject C, our SDT method was associated with a decrease in the growth rate of the tumor, likely due to the combination strategy. Finally, in subject D, a calcified mass inside the tumor and a metastatic lung tumor disappeared by 49 days post-SDT, and an increase in urinary frequency was observed by veterinarians in a subjective assessment. The impacts on the calcified mass were likely caused by the action of HIFU, even though its output power was one order of magnitude lower than that used in conventional HIFU therapy. The effects on the metastatic lung cancer were unanticipated, but may have been caused by the anticancer activity of the drug or the immune response induced by SDT. Similarly, PDT, which combines a photosensitizer with a laser, has been used for local treatment in the same manner as SDT, and the PDT-induced anti-tumor immunity has been reported (Castano et al., 2006). This outcome indicates that our SDT method could have potential not only as a local treatment but also as a full-body treatment.

Our SDT system utilizes a specific HIFU irradiation sequence consisting of a trigger pulse and heating wave to generate and maintain cavitation bubbles (Yoshizawa et al., 2017), which induce a cytotoxic effect. In conventional ultrasound diagnosis, a contrast agent with a microbubble is used intravenously to obtain a clear ultrasound image. In recent studies, such microbubbles have been used for new diagnostic and therapeutic approaches. Willmann et al. proposed a molecular-targeted ultrasound imaging technique for breast cancer, in which a microbubble targeted to the kinase insert domain receptor (KDR) that specifically accumulates in breast cancer, returning a strong signal on a contrast-mode image (Willmann et al., 2017). In addition, focused ultrasound in combination with gas-filled microbubbles was applied to transiently disrupt the blood–brain barrier (BBB) for enhanced drug delivery (Åslund et al., 2017). However, intravenous injection of microbubbles carries the risk of effects on healthy blood vessels and cells in the path from the body surface to the target point when ultrasound irradiation. By contrast, our easy-to-use HIFU system generates cavitation bubbles locally when needed and is safer than intravenous microbubble administration. Regarding the effect of HIFU on temperature rise, it was estimated to be low based on the temperature increase in the study of Jimbo et al., that was calculated to be about 5–6 K in gel-phantom experiments with the same HIFU configuration as ours (Jimbo et al., 2016). In our clinical trial, we expected an even lower temperature rise due to the energy loss at the boundaries of different tissues and the cooling effect caused by blood flow in the live body. We believe that there are no possible serious adverse events and noticeable thermal effects due to temperature increase during therapy.

Based on the results presented herein, our SDT approach can be safely performed on pet dogs suffering from spontaneous tumors, including osteosarcoma, hepatocellular cancer, and prostate cancer. SDT offers a potential cure for some cancers based on its multiple effects from solo HIFU, solo NC-6300, and the combination of both agents, as well as possible secondary effects, such as on immune activity. However, there are some potential limitations of this study. The treatment condition in this canine clinical trial was not the exact same as that in humans, and we need to confirm the procedure and safety of our proposed SDT without anesthesia. In addition, an assured HIFU irradiation requires some skills such as an identification of the tumor's placement on a US image, understanding of HIFU irradiation characteristics, and a risk prediction for doctors; in this study, the gastroenterology physician of Tokyo Medical University, who has participated in more than 200 cases of solo HIFU therapy, contributed to the study. To perform SDT stably and efficiently, further techniques, such as image processing and a robot control, would be required. Further, we plan to continue research into characterizing the mechanism of action of our SDT approach and to conduct an investigator-initiated clinical trial to evaluate the efficacy of SDT. In terms of canine cancer care, few standards have been established for standard of care, unlike in humans (Paoloni and Khanna, 2008). Thus, this therapy has the potential to become a standard therapy and contribute to the treatment of both pet and human cancer.

## Ethics Statement

All therapies were performed under the guidelines of the Animal Use Committee of Tottori University and Tokyo Women's Medical University. The protocol was approved by the Committee on the Ethics of Animal Experiments of Tottori University (Approval Number: H27-001) and Tokyo Women's Medical University (Approval Number: AE16-143). Before treatment, informed consent was obtained from the guardians of the animals.

## Author Contributions

YH contributed to coordination of SDT. MM contributed to management of treatment. YK contributed to HIFU irradiation planning. JO contributed to mechanical management. SI contributed to drug dosage selection. YO contributed to animal care and implementation of HIFU. HIsh contributed to animal care. SY contributed to setup of the HIFU transducer. SU contributed to setup of the HIFU transducer. TU contributed to setup of the HIFU robot. ST contributed to setup of the ultrasonic diagnostic equipment. AS contributed to ultrasound image diagnosis. KT contributed to drug supply and dosage selection. KM contributed advice on electrical safety and the HIFU system. HIse contributed medical advice on canine therapy. NN and KK contributed advice on the SDT mechanism. YM contributed to the generation of the SDT concept using an anticancer micelle (NC-6300) and HIFU, as well as total therapy management.

### Conflict of Interest Statement

MM was employed by MakeWay Merger Company. TU was employed by DENSO Corporation. ST was employed by Hitachi, Ltd. KT was employed by Kowa Company. The remaining authors declare that the research was conducted in the absence of any commercial or financial relationships that could be construed as a potential conflict of interest.

## Supplementary Material

Video S1 Improvement in walking before and after SDT (session 1).

Video S2 Improvement in walking before and after SDT (session 3).
